# Fecal microbiome transplantation and tributyrin improves early cardiac dysfunction and modifies the BCAA metabolic pathway in a diet induced pre-HFpEF mouse model

**DOI:** 10.3389/fcvm.2023.1105581

**Published:** 2023-02-08

**Authors:** Jomana Hatahet, Tyler M. Cook, Raiza R. Bonomo, Nadia Elshareif, Chaitanya K. Gavini, Chelsea R. White, Jason Jesse, Virginie Mansuy-Aubert, Gregory Aubert

**Affiliations:** ^1^Department of Cell and Molecular Physiology, Stritch School of Medicine, Loyola University Chicago, Maywood, IL, United States; ^2^Department of Biomedical Science, University of Lausanne, Lausanne, Switzerland; ^3^Division of Cardiology, Department of Internal Medicine, Stritch School of Medicine, Loyola University Chicago, Maywood, IL, United States

**Keywords:** heart failure with preserved ejection fraction, gut microbiome, short chain fatty acids, branched chain amino acids (BCAAs), obesity

## Abstract

More than 50% of patients with heart failure present with heart failure with preserved ejection fraction (HFpEF), and 80% of them are overweight or obese. In this study we developed an obesity associated pre-HFpEF mouse model and showed an improvement in both systolic and diastolic early dysfunction following fecal microbiome transplant (FMT). Our study suggests that the gut microbiome-derived short-chain fatty acid butyrate plays a significant role in this improvement. Cardiac RNAseq analysis showed butyrate to significantly upregulate *ppm1k* gene that encodes protein phosphatase 2Cm (PP2Cm) which dephosphorylates and activates branched-chain α-keto acid dehydrogenase (BCKDH) enzyme, and in turn increases the catabolism of branched chain amino acids (BCAAs). Following both FMT and butyrate treatment, the level of inactive p-BCKDH in the heart was reduced. These findings show that gut microbiome modulation can alleviate early cardiac mechanics dysfunction seen in the development of obesity associated HFpEF.

## Introduction

Heart failure with preserved ejection fraction (HFpEF) continues to be one of the most hypercritical cardiovascular diseases in the United States that requires immediate intervention (1, 2). It accounts for more than half of heart failure patients but has only one guideline-directed treatment, the sodium-glucose cotransporter 2 (SGLT2) inhibitors (3). The pathophysiology of HFpEF is complex, and this syndrome has been increasingly characterized as heterogeneous. Better phenotyping of patients into common pathophysiological groups has been proposed as a tool to treat HFpEF better (4). Obesity is the main driver for the pathogenesis of HFpEF with more than 80% of HFpEF patients being overweight or obese, making obesity associated HFpEF a specific pathological entity (4, 5). To date, obesity associated HFpEF studies involve the induction of systemic inflammation which increases reactive oxygen species (ROS) and oxidative stress, increases collagen deposition, limits nitric oxide (NO) bioavailability, and decreases protein kinase G (PKG) activity. This ultimately leads to cardiomyocyte hypertrophy, left ventricular (LV) stiffness, fibrosis, and the development of diastolic dysfunction and exercise intolerance (5–10). The presence of a new pathological identity termed “pre-HFpEF” has been recently identified where patients have no signs and symptoms of heart failure, they have normal ejection fraction of >50%, however, they show structural abnormalities to their hearts that resemble those found in clinical HFpEF, such as LV hypertrophy (11). It is important to understand and act on early cardiac changes observed in pre-HFpEF prior to transition to clinical HFpEF therefore our study focuses on the pre-HFpEF stage.

Among the shared risk factors between cardiovascular diseases and obesity is the accumulation of circulating branch chain amino acid (BCAA) and its decreased metabolism (12, 13). Impaired cardiac BCAA metabolism is associated with cardiac insulin resistance and the development of cardiovascular diseases (13–16). This occurs due to a decrease in the levels and activity of branched-chain alpha-keto acid dehydrogenase (BCKDH) complex that catalyzes the first irreversible step in the catabolism of branched chain amino acids (BCAAs). The increase in the levels of inactive p-BCKDH has been attributed to the downregulation of *ppm1k* gene encoding protein phosphatase 2C (PP2Cm) (17, 18). The aforementioned changes were observed once the HFpEF pathophysiology is installed, and more studies are necessary to discover the early mechanism underlying these changes.

It is well established that diet-induced obesity (DIO) mouse models are coupled with gut microbiome imbalance. Western diet (WD)-fed mice (high fat, high carbohydrate, low fiber) have significant reduction in their microbiome diversity and composition compared to control mice (19, 20), as well as a significant decrease in the short chain fatty acid (SCFA) butyrate producing bacteria *Lactobacillus* and *Lachnospiraceae* (19, 20). Interestingly, microbial metagenome and metabolomic analysis found significant reduction in butyrate producing bacteria in patients with chronic heart failure with reduced ejection fraction (HFrEF) (21). More recently, microbiome DNA sequencing analysis in HFpEF patients showed significant alterations in gut microbiome composition as well as a reduction in SCFA producing bacteria compared to control groups (22, 23). Butyrate, a microbiome-secreted SCFA, was shown to prevent cardiac hypertrophy progression in a pressure overload model of cardiac hypertrophy (24), and was found to improve cardiac function and ventricular arrhythmia in rats after myocardial injury (25).

The molecular mechanisms linking the gut microbiome imbalance, the circulating SCFAs, particularly butyrate, and the development of HFpEF are still unknown. Studies to date have not deciphered whether the microbiome imbalance observed in HFrEF and HFpEF patients is a secondary finding related to poor gut hemodynamic or the primary driver of the cardiac physiopathology. We investigated the effect of gut microbiome modulation using fecal microbiome transplantation (FMT) in the early stages of obesity associated HFpEF. We developed an obesity associated model of early cardiac dysfunction (pre-HFpEF) and focused on the early asymptomatic changes in cardiac mechanics that occur in the absence of increased intracardiac pressure.

Our study provides an insight on the potential role of gut microbiome and its metabolite butyrate in the early stage of obesity associated HFpEF and identifies the branched chain amino acids (BCAAs) metabolic pathway as a possible link between microbiome imbalance, obesity, and heart failure. These results open a new avenue not only for therapy but also for the prevention of HFpEF development and progression.

## Results

### Mice fed western diet (WD) developed early systolic and diastolic dysfunction consistent with pre-HFpEF

**FIGURE 1:**
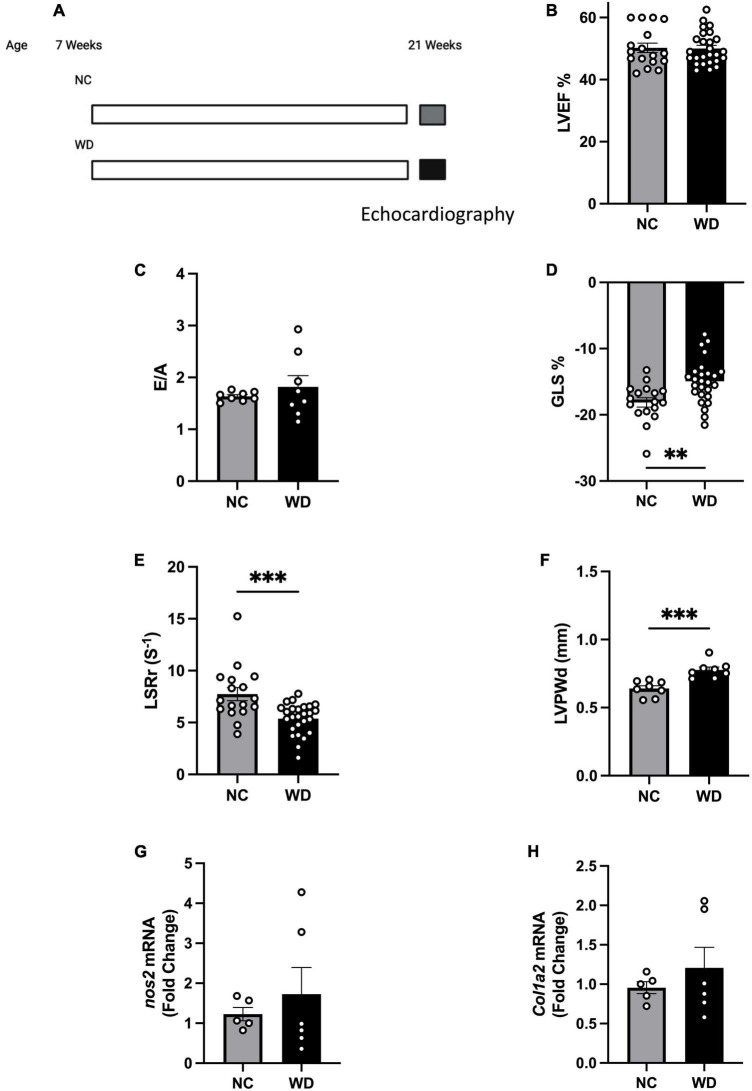
Mice fed western diet (WD) developed early systolic and diastolic dysfunction and cardiac hypertrophy. **(A)** Experimental paradigm. Normal chow group was fed NC (Teklad LM-485), while the western diet group was fed WD (TD88137, Teklad Diets; 42% kcal from fat, 34% sucrose by weight, and 0.2% cholesterol total; Envigo) for 14 weeks, starting at 7 weeks of age. Echocardiography measurements were performed at 21 weeks of age, **(B)** echocardiography measurement of left ventricle ejection fraction (LVEF), **(C)** ratio between early to atrial diastolic trans mitral flow velocity (E/A), **(D)** global longitudinal strain (%GLS), **(E)** longitudinal strain rate reverse (LSRr) (s^– 1^), **(F)** left ventricle posterior wall diameter during diastole (mm), **(G,H)** mRNA levels of *nos2* and *col1a2* in hearts of mice from different experimental groups, respectively. Statistical analysis was done using unpaired student’s *t*-test. Data are mean ± S.E.M. (*p < 0.05, ^**^p < 0.005, and ^***^p < 0.0005).

To study obesity associated pre-HFpEF, we developed a model of diet-induced obesity and assessed cardiac function. C57BL/6J mice were placed on WD for 14 weeks (Figure 1A) and compared to their littermates on normal chow (NC). We performed echocardiography measurements to investigate changes in cardiac function. As expected in a pre-HFpEF model, mice on WD had no change in their left ventricular ejection fraction (LVEF) (Figure 1B), and the ratio between peak velocity blood flow from left ventricular relaxation in early diastole (the E wave) to peak velocity flow in late diastole caused by atrial contraction (the A wave) (E/A ratio measurement) (Figure 1C) compared to NC mice. Mice fed a WD had significant decrease in global longitudinal strain (%GLS) (3.190% ± 0.9887) and longitudinal strain rate reverse peak (LSRr) (−2.363 s^–1^ ± 0.6213) indicating, respectively early signs of systolic and diastolic dysfunction (unpaired *t*-test, *P* = 0.0025, *P* = 0.0005, respectively) (Figures 1D, E). WD mice showed significant increase in their left ventricle posterior wall thickness during diastole (0.1368 mm ± 0.02956) (unpaired *t*-test, *P* = 0.0004) (Figure 1F) indicating the development of LV hypertrophy. As an early model to assess obesity associated HFpEF development, mice on WD had no significant increase in nitrosative stress or cardiac fibrosis, two players in the pathogenesis of HFpEF, indicated by no changes in *nos2* and *col1a2* (Figures 1G, H) expression levels. Taken together, our data show obese mice to have normal ejection fraction, early diastolic and systolic dysfunction, LV hypertrophy, with an absence of nitrosative stress and fibrosis. All of which are consistent with a diet-induced obesity pre-HFpEF phenotype.

### Fecal matter transplantation (FMT) improved early systolic and diastolic dysfunction and cardiac hypertrophy in obese pre-HFpEF mice

**FIGURE 2:**
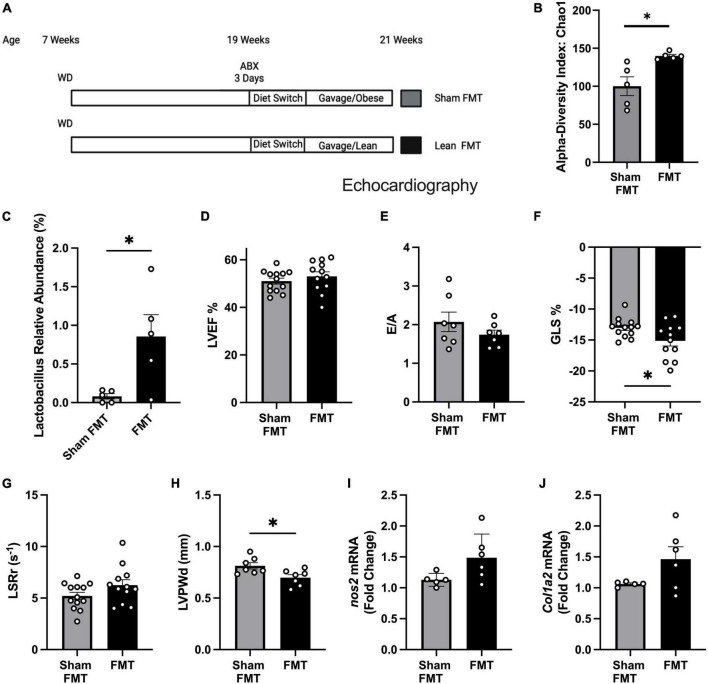
Fecal microbiome transplant (FMT) treatment improved diastolic dysfunction and cardiac hypertrophy in obese pre-HFpEF mice. **(A)** Experimental paradigm, C57BL/6J mice fed WD for 12 weeks, followed by broad-spectrum antibiotic treatment for 3 days mice were gavaged daily for 2 weeks with feces either from obese mice (sham FMT group) or from lean mice (FMT group), **(B)** alpha diversity index (Chao1), **(C)** *Lactobacillus* relative abundance (%), **(D)** representative echocardiography measurement of left ventricle ejection fraction (LVEF), **(E)** ratio between mitral E wave to A wave (E/A), **(F)** global longitudinal strain (%GLS), **(G)** longitudinal strain rate reverse (LSRr) (s^– 1^), **(H)** left ventricle posterior wall diameter during diastole (mm). **(I,J)** mRNA levels of *nos2* and *col1a2* in hearts of mice from different experimental groups. Statistical analysis was done using unpaired student’s *t*-test. Data are mean ± S.E.M. (*p < 0.05, ^**^p < 0.005).

To test whether gut microbiome alteration can reverse or delay pre-HFpEF progression, we treated obese pre-HFpEF mice with an established protocol (19) that includes an antibiotics treatment for 3 days, to deplete the gut microbiome followed by 5 days of diet switch to colonize the gut with bacteria that grows in NC conditions. Then fecal matter transplantations (FMT) were performed from either obese mice (Sham FMT) or lean mice (FMT) for 2 weeks (Figure 2A). 16S rRNA sequencing of fecal content showed FMT treated mice to have increased microbiome diversity compared to mice treated with sham FMT, indicated by higher α-diversity index (39.76 ± 12.48) (unpaired *t*-test, *P* = 0.0129) (Figure 2B). We had previously found that WD depletes *Lactobacillus* in both fecal pellets and cecal contents (19). Strikingly, FMT was able to increase butyrate-producing bacteria *Lactobacillus* abundance (0.7753% ± 0.2836) [Figure 2C (relative abundance), Supplementary Figure 1A (absolute abundance), Supplementary Datasheet 1]. Sparse Correlations for Compositional data (SparCC) network analysis identified *Lactobacillus* as a key marker of the FMT microbiome landscape as its presence was correlated with other genera (Supplementary Figure 1B) that were significantly altered between sham FMT and FMT groups (Supplementary Table 1). We measured cardiac function with echocardiography. LVEF (Figure 2D) and E/A (Figure 2E) did not change after WD nor FMT. We found that mice receiving FMT from lean mice had significant improvement in their global longitudinal strain (−2.105% ± 0.9206) (unpaired *t*-test, *P* = 0.0318) (Figure 2F). The trend in longitudinal strain rate reverse peak (LSRr) improvement did not reach statistical significance (unpaired *t*-test, *P* = 0.1075) (Figure 2G). The left ventricle posterior wall thickness (LVPWd) was significantly decreased by FMT treatment (−0.1151mm ± 0.04217) (unpaired *t*-test, *P* = 0.0182) (Figure 2H). In addition, we found no changes in *nos2* and *col1a2* (Figures 2I, J) expression levels between sham FMT and FMT groups. These data indicate that modulation of gut microbiota induced by FMT from lean mice to obese mice improves early systolic and diastolic dysfunction, as well as LV hypertrophy in obese pre-HFpEF mice. Overall, these findings suggest a key role for SCFA producers *Lactobacillus* in the reversal of diet-induced obesity pre-HFpEF.

### Tributyrin treatment improved early cardiac dysfunction and cardiac hypertrophy in obese pre-HFpEF mice

**FIGURE 3:**
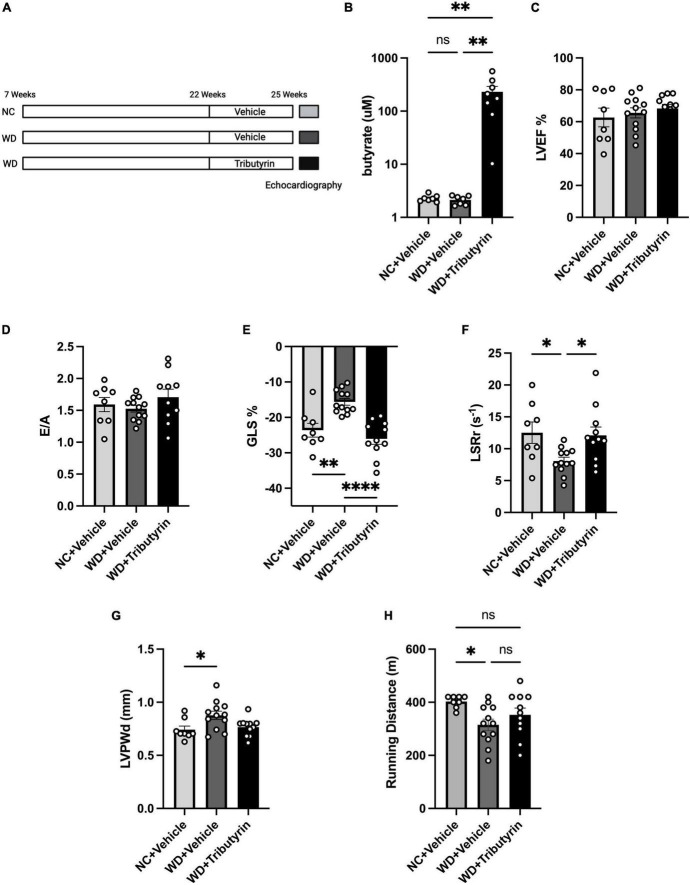
Tributyrin treatment improved early cardiac dysfunction and cardiac hypertrophy in obese pre-HFpEF mice. **(A)** Experimental paradigm, C57BL/6J fed WD or NC for 15 weeks were treated with Tributyrin using needle feeding for 3 weeks, followed by echocardiography and exercise exhaustion test. **(B)** Circulating levels of butyrate in serum (μM), **(C)** echocardiography measurements of LVEF (%), **(D)** ratio between mitral E wave to A wave (E/A), **(E)** global longitudinal strain (GLS) (%), **(F)** longitudinal strain rate reverse (LSRr) (S^– 1^), **(G)** left ventricle posterior wall diameter (mm). **(H)** Running distance during exercise exhaustion test (m). Statistical analysis was done using one-way ANOVA followed by Tukey’s multiple comparison test. Data are mean ± S.E.M. (*p < 0.05, ^**^p < 0.005, and ^****^p < 0.00005), (*n* = 8–12 per group).

We identified *Lactobacillus*, a SCFA-producer, as a key marker of the FMT microbiome landscape. We also recently showed that circulating serum levels of the SCFA butyrate were significantly increased in obese mice after FMT (19). We wanted then to investigate whether cardiac function improvements in obese pre-HFpEF mice after FMT treatment can be recapitulated by the SCFA butyrate. We previously tested various routes of butyrate treatment, doses, and drug compounds and observed an increase in circulating butyrate in blood of tributyrin (three butyrate molecules with a glycerol backbone) -treated mice (19). We then treated WD-fed mice with tributyrin (5 g/kg body weight) or vehicle for 3 weeks (Figure 3A). We measured the circulating levels of butyrate in Tributyrin treated mice using mass spectrometry and we found it to be significantly increased compared to vehicle treated mice (228.5 μM ± 55.08) (one-way ANOVA with repeated measures followed by Tukey’s multiple comparison test, *P* < 0.005) (Figure 3B). Next, we measured changes in cardiac function with echocardiography and similar to our findings after FMT treatment, LVEF and E/A did not change between NC, WD and WD + Tributyrin treated mice (Figures 3C, D). As seen in Figure 1, mice on WD had significant decrease in their%GLS compared to NC mice (8% ± 2.108), and this change was significantly improved with tributyrin treatment (−10.43% ± 1.928) (one-way ANOVA with repeated measures followed by Tukey’s multiple comparison test, *P* < 0.0001) (Figure 3E). We also found Tributyrin treatment to significantly improve LSRr in WD fed mice compared to vehicle treatment (4.002 s^–1^ ± 1.574) (one-way ANOVA with repeated measures followed by Tukey’s multiple comparison test, *P* < 0.05) (Figure 3F) indicating effectiveness of tributyrin treatment in eliminating early signs of systolic and diastolic dysfunction. Additionally, changes in LVPWd observed in WD fed mice compared to NC were eliminated after tributyrin treatment (one-way ANOVA with repeated measures followed by Tukey’s multiple comparison test, *P* < 0.05) (Figure 3G). Severe exercise intolerance is very common in obese and HFpEF patients (9, 10); therefore, we performed an exercise exhaustion test. Mice fed WD had significantly decreased running distance compared to NC (−87.50 m ± 31.61), and this difference was eliminated by tributyrin treatment (37.73 m ± 28.91) (one-way ANOVA with repeated measures followed by Tukey’s multiple comparison test, *P* < 0.05) (Figure 3H). These results suggest that FMT and butyrate share common mechanisms in improving early cardiac function and LV hypertrophy in obese pre-HFpEF mice.

### Tributyrin treatment increased *ppm1k* transcript in the heart

**FIGURE 4:**
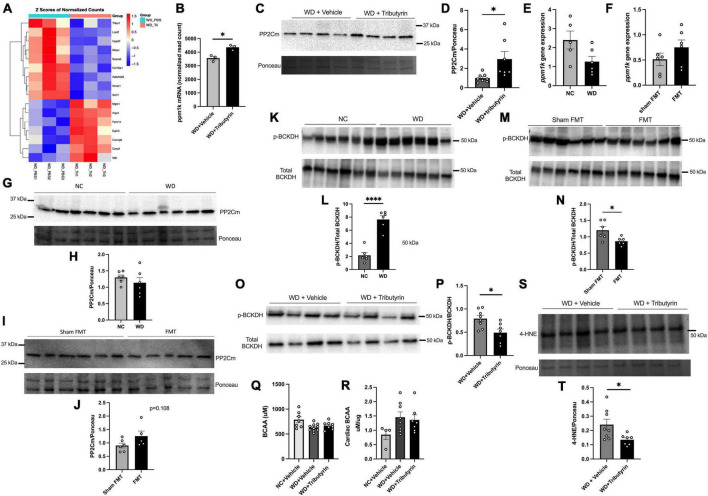
Tributyrin alters transcripts in the heart. **(A)** Topmost regulated transcripts after Tributyrin treatment in the heart (WD, WD + Tributyrin needle fed mice), FDR-adjusted *P* < 0.1 (n = 3 per group) (Supplementary Datasheet 2). **(B)** *ppm1k* mRNA normalized read count in WD and WD + Tributyrin treated mice (FDR-adjusted *P* = 0.049515631) **(C)** Immunoblot images of PP2Cm and total ponceau staining from hearts of WD fed mice treated with Tributyrin or vehicle. **(D)** Densitometric analysis of the ratio of PP2Cm to total protein staining bands, (*n* = 7–8 per group). **(E)** mRNA levels of *ppm1k* in heart from NC and WD fed mice (*n* = 5–6 per group). **(F)** mRNA levels of *ppm1k* in heart from sham FMT and lean FMT treated mice (*n* = 6 per group). **(G)** Immunoblot images of PP2Cm and total protein staining from NC and WD fed mice. **(H)** Densitometric analysis of the ratio of PP2Cm to total protein staining bands (*n* = 6 per group). **(I)** Immunoblot images of PP2Cm and total protein staining from sham FMT and FMT treated mice. **(J)** Densitometric analysis of the ratio of PP2Cm to total protein staining bands (*n* = 5–6 per group). **(K)** Immunoblot images of p-BCKDH, total BCKDH and total protein staining from hearts of NC and WD fed mice. **(L)** Densitometric analysis of the ratio of p-BCKDH/BCKDH protein bands (*n* = 6 per group). **(M)** Immunoblot images of p-BCKDH, total BCKDH and total protein staining from hearts of WD fed mice treated with sham FMT or FMT. **(N)** Densitometric analysis of the ratio of p-BCKDH/BCKDH protein bands (*n* = 6 per group). **(O)** Immunoblot images of p-BCKDH, total BCKDH and total protein staining from hearts of WD fed mice treated with Tributyrin or vehicle. **(P)** Densitometric analysis of the ratio of p-BCKDH/BCKDH protein bands (*n* = 7–8 per group). **(Q)** Serum BCAA levels in NC, WD and WD + Tributyrin treated mice (*n* = 8–10). **(R)** Cardiac BCAA levels in NC, WD and WD + Tributyrin treated mice normalized to total protein levels (n = 4–8). **(S)** Immunoblot images of 4-HNE and total protein staining from hearts of WD fed mice treated with Tributyrin or vehicle, **(T)** Densitometric analysis of the ratio of 4-HNE to total protein staining protein bands. Statistical analysis was done using unpaired student’s *t*-test. Data are mean ± S.E.M. (*p < 0.05), (*n* = 7–8 per group). Statistical analysis was done using unpaired student’s *t*-test and one-way ANOVA followed by Tukey’s multiple comparison test. Data are mean ± S.E.M. (*p < 0.05, ^****^p < 0.00005).

To better understand the molecular mechanisms leading to improvements in early cardiac function in obese pre-HFpEF mice following Tributyrin treatment, we performed cardiac RNA sequencing analysis of WD and WD + Tributyrin treated mice. Out of 23,564 transcripts identified, using an FDR (false discovery rate) of 10%, we found 34 transcripts to be altered with tributyrin treatment (Figure 4A and Supplementary Datasheet 2). Among the significantly altered genes, is the transcript protein phosphatase Mg2 + /Mn2 + dependent 1K (*ppm1k*). Tributyrin treated mice had a significant increase in *Ppm1k* transcript levels compared to WD treated mice (795.0 read count ± 175.7) (Figure 4B, false-discovery rate (FDR)-adjusted *P* < 0.1). *Ppm1k* encodes protein phosphatase 2C (PP2Cm), which in turn is responsible for the dephosphorylation and activation of branched-chain alpha-keto acid dehydrogenase (BCKDH) complex that catalyzes the first irreversible step in the catabolism of branched chain amino acids (BCAAs); L-leucine, L-valine, and L-isoleucine, and in turn improves BCAA metabolism (17, 26). In addition to the increase in *ppm1k* transcript levels, we found the encoded PP2Cm protein levels to be significantly increased (1.958 A.U. ± 0.7367) in the heart of WD + Tributyrin group compared to WD (unpaired *t*-test, *P* = 0.0197) (Figures 4C, D). Without butyrate treatment, *Ppm1k* mRNA level had a trend toward decreasing in WD-fed mice compared to NC and a trend toward increasing in lean FMT treated mice compared to sham-FMT (Figures 4E, F). Similarly, PP2Cm protein levels trended lower in WD-fed mice (Figures 4G, H) and higher in FMT compared to sham FMT treated mice (Figures 4I, J).

This further implies that improvements seen with FMT treatment share common mechanism with treatment with SCFA butyrate.

### Tributyrin treatment decreased the p-BCKDH inactive enzyme in the BCAA metabolism pathway

Impaired BCAA metabolism occurs due to a decrease in the levels and activity of BCKDH and an increase in the levels of inactive p-BCKDH and that has been attributed to the downregulation of *ppm1k* translated protein PP2Cm (17, 18). We found the protein levels of p-BCKDH to be significantly increased in WD fed mice compared to NC (5.477 A.U. ± 0.7178) (unpaired *t*-test, *P* < 0.0001) (Figures 4K, L). However, p-BCKDH was significantly blunted in the heart after FMT treatment (−0.3352 ± 0.1266) (unpaired *t*-test, *P* = 0.0380) (Figures 4M, N) as well as after tributyrin treatment (−0.3004 ± 0.1031) (unpaired *t*-test, *P* = 0.0121) (Figures 4O, P). The levels of circulating BCAAs in serum was not changed between WD and WD + Tributyrin in both male and female treated mice (Figure 4Q). To better assess the cardiac BCAA metabolism, we measured cardiac BCAAs concentration. We found no significant changes in cardiac BCAA between the three different groups (Figure 4R). BCAA catabolism defects are known to be associated with heart failure development due to increase in oxidative stress and reactive oxygen species (ROS) (3, 19–22, 27–29). Elevated ROS levels lead to lipid peroxidation, where unsaturated lipids are converted to lipid peroxides that generate highly reactive and damaging lipids such as 4-hydroxynonenal (4-HNE) (30, 31). Therefore, we investigated whether tributyrin treatment decreased oxidative stress in the heart by measuring the levels of 4-HNE. We found the levels of 4-HNE to be significantly reduced with tributyrin treatment in obese pre-HFpEF mice (−0.1064 ± 0.04307) (unpaired *t*-test, *P* = 0.0281) (Figures 4S, T). These findings indicate the potential role of butyrate in the BCAAs metabolism pathway by increasing the levels of PP2Cm, decreasing p-BCKDH, which activates the degradation of BCAAs and in turn decreases oxidative stress in the heart.

### Tributyrin upregulation of *ppm1k* occurred at least in part through histone deacetylase (HDAC) inhibition

**FIGURE 5:**
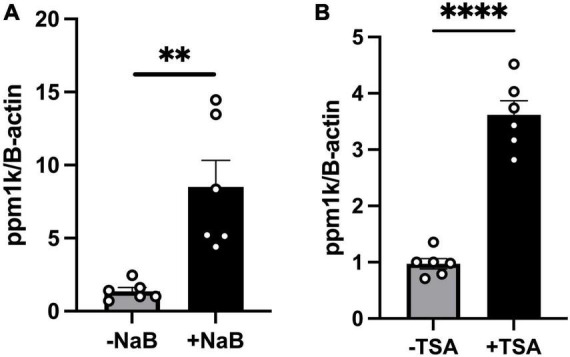
Tributyrin upregulation of *ppm1k* occurs at least in part through HDAC inhibition. **(A)** mRNA levels of *ppm1k* in C2C12 treated with 5mM sodium butyrate (NaB) or vehicle. **(B)** mRNA levels of *ppm1k* in C2C12 cells treated with 1 μM HDAC inhibitor Trichostatin A (TSA) or vehicle. Statistical analysis was done using unpaired student’s *t*-test. Data are mean ± S.E.M. (^**^p < 0.005, ^***^p < 0.0005, and ^****^p < 0.00005), (*n* = 6 per group).

It is well established that butyrate’s effects are mediated either through (a) binding to G-protein coupled receptors (GPCRs); GPR43 (FFAR2), GPR41 (FFAR3) which activates downstream signaling pathways (32–34), or (b) epigenetic regulation through its histone deacetylase (HDAC) inhibition activity, as well as activation of histone acetyltransferases (HATs) which increases histone acetylation and gene expressions (35, 36). To test whether butyrate upregulates *ppm1k* through binding to its GPCRs GPR41/43, we performed RNAscope *in situ* hybridization of GPR41/43 on cardiac tissue sections as done previously (20) from wild-type mice and we found no staining in the heart compared to nodose ganglia, a GPR41/43 positive tissue (Supplementary Figure 2). These data rule-out the involvement of the receptors in butyrate’s effect on heart function and hypertrophy. Next, we assessed whether HDAC inhibition may increase *ppm1k* mRNA level. We treated C2C12 cells with sodium butyrate and with the inhibitor of HDAC class I and II, Trichostatin A (TSA). We confirm the increase of *ppm1k* mRNA levels in butyrate treated cells compared to vehicle treatment (7.140 ± 1.838), and we observed that TSA also increased *ppm1k* expression compared to vehicle treatment (2.646 ± 0.2661) (unpaired *t*-test, *P* = 0.003, *P* < 0.0001, respectively) (Figures 5A, B). These data confirm that butyrate robustly regulates the levels of *ppm1k* in muscle cells and that butyrate HDAC inhibitor property may underlie these changes in gene expression.

## Discussion

The role of gut microbiome and its metabolites in the development of cardiovascular diseases (CVDs) is an emerging area of research (27, 37–39). In this study we investigate the role of gut microbiome and its metabolite butyrate in obesity associated pre-HFpEF mouse model that shows early signs of cardiac dysfunction and hypertrophy. Using a specific model of obesity associated pre-HFpEF we aimed at harnessing the early changes that link obesity to the development of HFpEF. There are several animal models to try to mimic and replicate human HFpEF (40). However, most try to better understand the late stage of disease development and their findings focus on culprit or adaptative changes. By using an early stage of HFpEF with one specific risk factor which is obesity, we believe this could better help understand the early derangements that ultimately lead to the development of HFpEF. Using fecal microbiome transplantation (FMT) from lean mice, we were able to modulate gut microbiome composition in obese pre-HFpEF mice. In addition, we found FMT to improve early cardiac dysfunction and cardiac hypertrophy. Indeed microbiome alteration is a hallmark of obesity but similar derangements were also recently found in heart failure patients (21) and especially HFpEF (22, 23). Microbiota imbalance could be considered as a signature of heart failure, however, in this study we show for the first time that correction of this imbalance can improve cardiac mechanics. This could help stipulate that this derangement is a cause of HFpEF development. Further study in germ free mice could help better understand this causality. FMT treatment of obese mice led to an increase in circulating levels of the SCFA butyrate (19), and increased the levels of butyrate-producing bacteria *Lactobacillus*, which implied that FMT’s improvement could occur due to SCFA butyrate. To confirm this, we treated obese pre-HFpEF mice with tributyrin, the triglyceride form of butyrate, and we were able to recapitulate FMT’s effects on cardiac function and hypertrophy. Additionally, tributyrin improved exercise capacity, one of the key markers of HFpEF development. This finding can be explained by the improvement of cardiac mechanics but also opens a new area of investigation on the ability of skeletal muscles to use butyrate as a substrate for energy production. Indeed some studies have shown the potential for ketone ester to improve performance (41, 42), and more recently supplementation with the SCFA producer *Lactobacillus plantarum* was shown to increase triathlete performance (43). WD-fed mice had no significant changes in their body weight after tributyrin treatment compared to vehicle treatment, which indicates that improvements seen in cardiac function and exercise capacity was not due to weight loss but possibly due to a direct effect of FMT and butyrate on the heart. Therefore, we performed cardiac RNAseq analysis and found significant increase in *ppm1k* which regulates the rate limiting enzyme involved in branched chain amino acids (BCAAs) catabolic pathway after Tributyrin treatment. BCAAs are essential amino acids that include Valine, Leucine, and Isoleucine. They play a key role in protein synthesis, cell signaling and metabolism (44, 45). Accumulation of BCAAs has been shown in obesity and several obesity-associated cardiovascular diseases, which occurs due to an impairment in their catabolic pathway (13, 15, 16). This occurs mainly due to a deficiency in the presence and activity of branched-chain α-keto acid dehydrogenase (BCKDH) which is controlled by the activity of protein phosphatase 2Cm (PP2Cm) (28). A decrease in PP2Cm correlates with a decrease in the levels and activity of the unphosphorylated BCKDH which further leads to deficiency in the degradation and catabolism of BCAAs (13, 17, 18). This suggests that butyrate’s improvement of early cardiac dysfunction, hypertrophy and exercise capacity could be mediated by enhancing the BCAAs catabolism pathway. We found the protein levels of PP2Cm and its encoding gene *ppm1k* to be significantly increased after Tributyrin treatment in obese pre-HFpEF. Concurrently, we found p-BCKDH to be significantly decreased in the heart. The regulation of *ppm1k* expression levels includes post-translational modifications such as phosphorylation, ubiquitination, and acetylation. Since butyrate is a well-known epigenetic regulator due to its histone deacetylation (HDAC) inhibition property, we tested whether butyrate’s increase in *ppm1k* expression was through HDAC inhibition. We found the pharmacological HDAC inhibitor, Trichostatin A (TSA), to replicate at least in part butyrate’s increase of *ppm1k* mRNA levels *in vitro*. This indicates that HDAC inhibition is one of the mechanisms by which butyrate increases *ppm1k* levels, but further work should be done to further understand the detailed effect of butyrate on *ppm1k* transcription. In addition, future work should be done to investigate whether butyrate can regulate *ppm1k* expression through activating HATs and increasing histone acetylation.

Although we found tributyrin treatment to increase PP2Cm and decrease p-BCKDH levels in the heart, we detected no change in the levels of serum or cardiac BCAAs. This is consistent with other findings of mice on HFD having no changes in their circulating BCAAs levels (46, 47). Tributyrin’s increase in BCAA catabolic enzymes suggests that its role in improving cardiac function and hypertrophy in WD-fed mice is independent of changes in circulating BCAAs levels. It is still possible that changes in BCAAs occur in a cell specific manner and small differences could not be accurately measured in circulating or whole organ measurements. One of the main effects of BCAAs catabolic pathway defects is the increase in reactive oxygen species (ROS) production (18, 29, 47). Elevated levels of ROS is a known characteristic of obesity and HFpEF (1, 7, 48). Therefore, we measured the levels of 4-hydroxynonenal (4-HNE), a highly reactive and damaging lipid that is produced from lipid peroxidation and a marker of oxidative stress (31). We found the levels of 4-HNE to be significantly reduced in the heart after Tributyrin treatment in obese pre-HFpEF mice. This finding could imply that Tributyrin’s effect on oxidative stress is through its change in PP2Cm and BCKDH expression, but future work would need to be done to measure the direct effect of increasing/decreasing PP2Cm and BCKDH expression and activity on ROS production.

Our study shows gut microbiome modulation and butyrate supplementation as a possible intervention to prevent the progression of obesity induced HFpEF. We identified the upregulation of BCAAs catabolic enzymes as a potential mechanism that mediates the effects of butyrate on cardiac function and hypertrophy, and a potential therapeutic target for the development of obesity associated HFpEF.

## Materials and methods

### Animals

Animal studies were conducted in accordance with recommendations in the Guide for the Care and Use of Laboratory Animals of the National Institutes of Health (49) and with the approval of the Loyola University Chicago Institutional Animal Care and Use Committee. Wild-type, C57BL/6J mice were obtained from Jackson Laboratories. Male and Female mice were fed NC (Teklad LM-485), while the experimental groups were fed WD (TD88137, Teklad Diets; 42% kcal from fat, 34% sucrose by weight, and 0.2% cholesterol total; Envigo).

### FMT treatment cohort

After 12 weeks of WD, mice were given antibiotics for 3 days followed by gavage treatment. Mice were switched to NC diet for the first 5 days of gavage, then continued with WD for the remainder of the 2 weeks treatment. Sham FMT mice were gavaged with feces from obese mice, while FMT mice were gavaged with feces from lean mice. Fresh feces from donors were collected the morning of gavage, homogenized *via* vortexing, and fecal slurry supernatant was collected avoiding solid particles.

### Tributyrin treatment cohort

At the end of week 15 on WD or NC, animals were needle-fed with Tributyrin (Sigma-Aldrich) or Vehicle 2 days on/2 days off for 3 weeks, at a dosage of 5g/kg of mice body weight.

### Echocardiography

Echocardiography was performed using a Visual Sonics Vevo 3100 system equipped with MX550D transducer (Visual Sonics). Anesthesia was induced by isoflurane and measurements were obtained from short-axis M-mode scans. Parameters collected include heart rate, stroke volume, LVEF, left ventricular fractional shortening, left ventricular end-diastolic diameter, left ventricular end-systolic diameter, left ventricular end-diastolic posterior wall, peak Doppler blood inflow velocity across the mitral valve during early diastole. At the end of the procedures all mice recovered from anesthesia without difficulties.

### Speckle-tracking echocardiography and strain analysis

B-mode traces were acquired and used to calculate global longitudinal strain and longitudinal strain rate reverse peak using Vevo Strain software (Visual Sonics) and a speckle-tracking algorithm.

### Exercise exhaustion test

After 3 days of pre-training for adjustment to the treadmill exercise, the exhaustion test was performed in all the experimental groups of mice. Mice ran on the treadmill staring at a speed of 5 m min^–1^ for 5 min. The treadmill speed was then increased by 1 m min^–1^ every minute until the mouse was exhausted. Continuation of running was encouraged by delivering a mild shock using an electric-stimulus grid. Exhaustion was defined as the inability of the mouse to return to running after 10 s of shock delivery. Running time was measured and calculated as total minutes ran by each mouse prior to exhaustion and running distance was calculated accordingly.

### RNA isolation, cDNA library construction, and illumina sequencing

Total RNA was extracted from mice hearts using the RNAeasy isolation kit (Qiagen). The stranded mRNA-seq was conducted in the Northwestern University NUSeq Core Facility. Briefly, total RNA examples were checked for quality using RINs generated from Agilent Bioanalyzer 2100. RNA quantity was determined with Qubit fluorometer. The Illumina Stranded mRNA Library Preparation Kit was used to prepare sequencing libraries from high-quality RNA samples (RIN > 7). The Kit procedure was performed without modifications. This procedure includes mRNA purification and fragmentation, cDNA synthesis, 3′ end adenylation, Illumina adapter ligation, library PCR amplification and validation. Lllumina HiSeq 4000 sequencer was used to sequence the libraries with the production of single-end, 50 bp reads at the depth of 20–25 M reads per sample. The quality of DNA reads, in FASTQ format, was evaluated using FastQC. Adapters were trimmed and reads of poor quality or aligning to rRNA sequences were filtered. The cleaned reads were aligned to the reference genome using STAR [Dobin et al. (50)]. Read counts for each gene were calculated using htseq-count [Anders et al. (51)]. Normalization and differential expression were determined using DESeq2 [Love et al. (52)]. The cutoff for determining significantly differentially expressed genes was an FDR-adjusted *p*-value less than 0.05. A pathway analysis was performed on both gene lists using GeneCoDis [Tabas-Madrid et al. (53); Nogales-Cadenas et al. (54); Carmona-Saez et al. (55)] to identify pathways that are enriched with genes that are upregulated and downregulated.

### Western blot analysis

Protein from frozen mouse hearts were prepared by lysis in ice-cold RIPA buffer (ThermoFisher, cat.89900) containing protease and phosphatase inhibitor (ThermoFisher, cat. A32959). Tissue was homogenized using bullet blender bead lysis kit (Next Advance), and protein concentrations were determined with Pierce BCA Protein Assay Kit (Thermofisher, cat. 23225). Proteins were separated by sodium dodecyl sulfate polyacrylamide gel electrophoresis (SDS-PAGE) on 4–15% gradient gel (Bio-Rad, cat.4561086) and transferred to PVDF membrane using iBlot 2 transfer system (ThermoFisher). Protein expression was measured by chemiluminescence using ChemiDoc imaging system (Bio-Rad). Proteins were detected with the following primary antibodies: BCKDH (ThermoFisher, cat.PA5-97248), phospho-BCKDH (S293) (abcam, ab200577), PP2Cm (abcam, ab135286), 4-hydroxynonenal (abcam, ab46545) (14, 56, 57).

### Cell culture and treatments

C2C12 mouse myoblasts cells were cultured in 1x DMEM (Corning) supplemented with 10% fetal bovine serum and 1% penicillin-streptomycin in a humidified 5% CO2 atmosphere at 37°C. Cells were treated with sodium butyrate (Sigma-Aldrich) dissolved in water for 6 h at 5 mM concentration. For HDAC inhibition cells were treated with Trichostatin A (Tocris) dissolved in DMSO for 6 h at 1 μM concentration (58).

### Reverse transcription quantitative polymerase chain reaction (RT-qPCR)

mRNA was isolated from C2C12 cells with different treatments using TRIZOL reagent (ref protocol). cDNA was synthesized from 1 μg of RNA using the High-Capacity cDNA Reverse Transcription Kit (Applied Biosystems). Quantitative PCR (qPCR) was performed in triplicate for each sample using diluted cDNA (1:10), SYBR Green (Roche, Cat.04913850001), and 10 μM of forward and reverse primers specific for *ppm1k* (F: GAGTTATGCCCACCTGTCTGCA, R: CT GTCTCCAACACTGGCTACCA) and β*-actin* (F: ACCTTCTAC AATGAGCTGCG, R: CTGGATGGCTACGTACATGC). Samples were cycled 50 times as following (95°C 15 s, 60°C 1 min, measure fluorescence), using CFX96 Real-Time System (Bio-Rad, Cat.1855196). PCR amplification was followed by a melt curve analysis to verify uniformity of amplicon product. Gene expression was calculated and quantified relative to the housekeeping gene β-actin using the ΔΔCt method.

### 16S sequencing and diversity analysis

As described previously (19, 20) cecal contents were collected and DNA extracted using the QIAamp Powerfecal DNA Kit (Qiagen). qPCR was performed with universal 16S primers. The Loyola Genomic Core performed PCR of 16S rRNA V4-5 regions sequenced by the Illumina HiSeq4500 platform, as done previously (59);16S sequences were aligned using the Silva Taxonomy Annotation, and files were uploaded to Microbiome Analyst for analysis (60) for analysis.^1^

### Butyrate and BCAA serum measurements

Blood was isolated from mice, after decapitation under anesthesia, collected in Sarstedt microvette blood collection tubes, and centrifuged at 2,000 × *g* for 10 min for serum collection. The quantification and analysis of serum butyrate and BCAA was performed by the Mass Spectrometry Core in Research Resources Center of University of Illinois at Chicago. Serum samples were stored in the −80°C freezer prior to use and were thawed in water bath for 30 s. 10 ul of serum sample was taken for deproteinization and 40 ul methanol (MeOH) was added before vortexing for 2 min. The samples were incubated at 4°C for 30 min and vortexed again thoroughly. The samples were then centrifuged at 14,000rpm for 10 min and 30 ul of the supernatant was taken for derivatization. For derivatization, 30 ul of each standard solution or sample supernatant was mixed with 15 ul of 200 mM 3-NPH in 50% aqueous MeOH and 15 ul of 120 mM EDC in the same solution. The reaction was allowed to proceed for 30 min at 40°C. The reaction mix was then diluted with 350 ul of 10% MeOH. LC/MS analysis was performed on AB SCIEX 6500 QTRAP coupled with Agilent 1,290 UPLC system.

The LC/MS data files were processed using the AB Sciex MultiQuant software.

### Cardiac BCAA measurement

Measurements were done following the BCAA colorimetric Assay protocol (BioVision, cat#K564). BCAA concentrations were normalized to total protein measured using BCA protein assay kit (ThermoFisher, cat#23225).

### *In situ* hybridization

*In situ* hybridization for GPR41 (ACD Probe Cat#) GRP43 (ACD Probe#) was done as described previously (20) using a combination of chromogenic RNAScope (FastRed) and GFP immunohistochemistry.

## Data availability statement

The data discussed in this publication have been deposited in NCBI’s Gene Expression Omnibus (61) and are accessible through GEO Series accession number: GSE221195 (https://www.ncbi.nlm.nih.gov/geo/query/acc.cgi?acc=GSE221195).

## Ethics statement

This animal study was reviewed and approved by Loyola University Chicago Institutional Animal Care and Use Committee.

## Author contributions

JH and TC designed the experiments, performed the experiments, analyzed the data, wrote the manuscript, and reviewed the manuscript. RB designed the experiments, performed the experiments, and reviewed the manuscript. NE, CG, CW, and JJ performed the experiments and reviewed the manuscript. VM-A and GA designed the experiments, analyzed the data, wrote the manuscript, and reviewed the manuscript. All authors contributed to the article and approved the submitted version.

## Footnotes

1
https://www.microbiomeanalyst.ca/

## Supplementary material

Supplementary Figure 1 **(A)** Log-transformed of *Lactobacillus* relative abundance in cecal contents from mice treated with sham FMT or lean FMT (*n* = 3–5). **(B)** SparCC analysis (correlation threshold > 0.5, *p* < 0.05) of 16S sequencing of cecal contents after sham FMT or lean FMT treatments.

Supplementary Figure 2 Chromogenic *in situ* hybridization staining of **(A)** *ffar2* mRNA (green) and **(B)** *ffar3* mRNA (green) in heart sections. **(C)** Chromogenic *in situ* hybridization staining of *ffar3* mRNA (green) in nodose ganglia (NG).

Supplementary Table 1 Summary table of DEseq2 analysis, data transformed using relative log expression.

Supplementary Table 2 Topmost regulated transcripts after Tributyrin treatment in the heart (WD, WD + Tributyrin needle fed mice), FDR-adjusted *P* < 0.1 (*n* = 3 per group).
