# Supplementary figures and images for: Fecal microbiome transplantation and tributyrin improves early cardiac dysfunction and modifies the BCAA metabolic pathway in a diet induced pre-HFpEF mouse model
Source: Front Cardiovasc Med. 2023 Feb 8;10:1105581. doi: 10.3389/fcvm.2023.1105581 (PMC9944585; doi:10.3389/fcvm.2023.1105581)

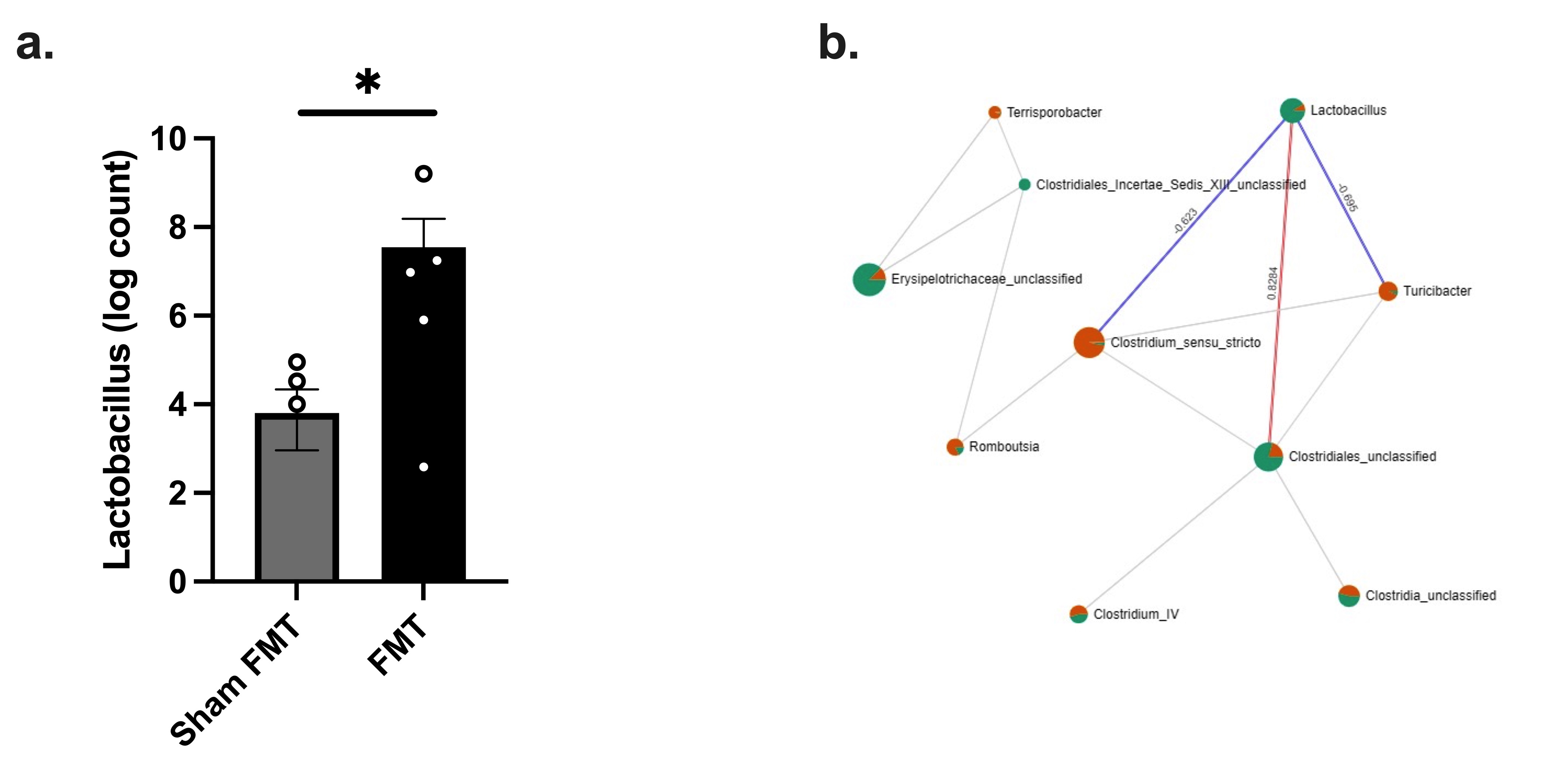

Supplement: Supplementary Figure 1 — (A) Log-transformed of Lactobacillus relative abundance in cecal contents from mice treated with sham FMT or lean FMT (n = 3–5). (B) SparCC analysis (correlation threshold > 0.5, p < 0.05) of 16S sequencing of cecal contents after sham FMT or lean FMT treatments. [file Image_1.JPEG]

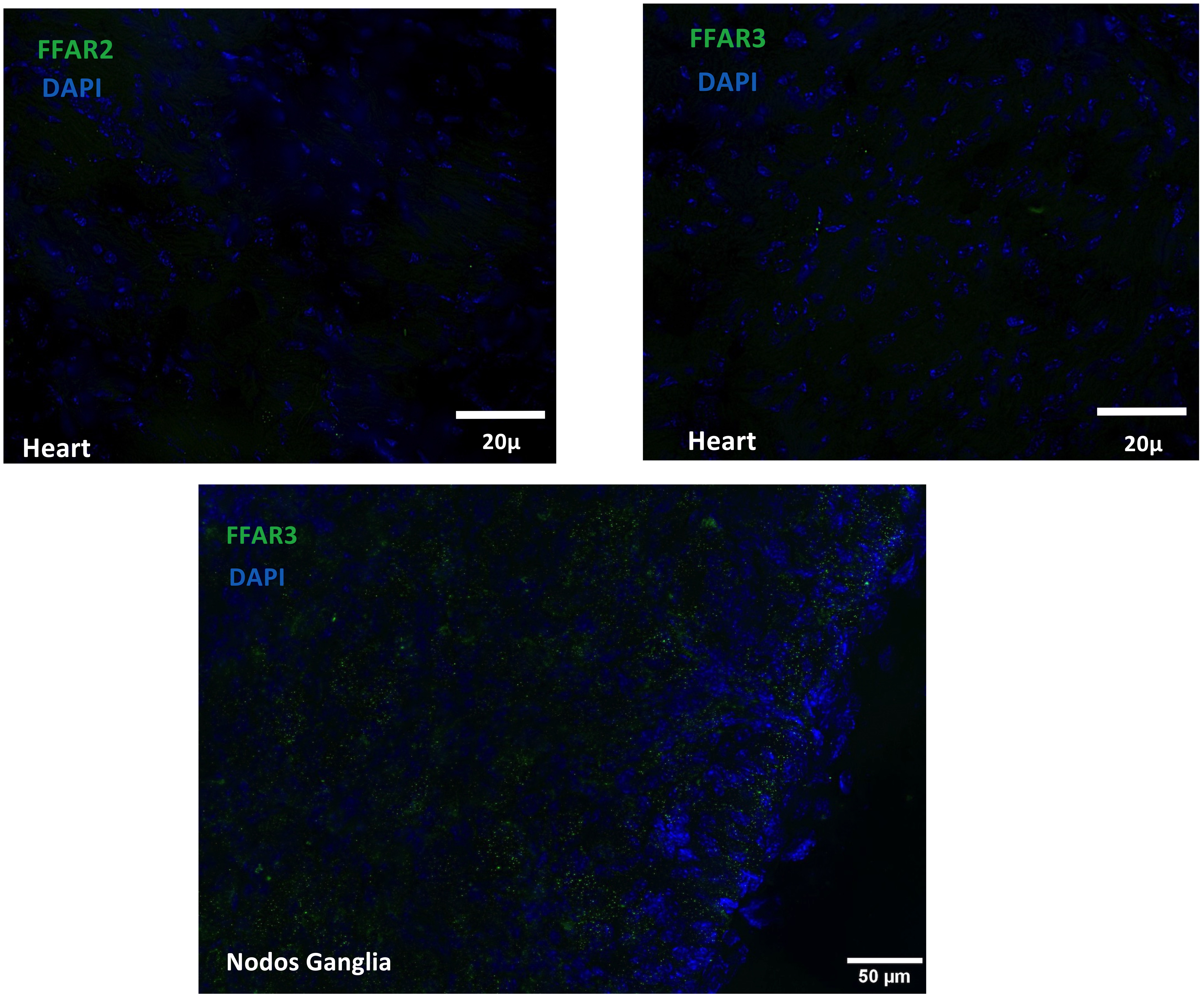

Supplement: Supplementary Figure 2 — Chromogenic in situ hybridization staining of (A) ffar2 mRNA (green) and (B) ffar3 mRNA (green) in heart sections. (C) Chromogenic in situ hybridization staining of ffar3 mRNA (green) in nodose ganglia (NG). [file Image_2.jpg]
