# Supplementary material for: Fecal microbiome transplantation and tributyrin improves early cardiac dysfunction and modifies the BCAA metabolic pathway in a diet induced pre-HFpEF mouse model
Source: Front Cardiovasc Med. 2023 Feb 8;10:1105581. doi: 10.3389/fcvm.2023.1105581 (PMC9944585; doi:10.3389/fcvm.2023.1105581)
Supplement: Supplementary Table 1 — Summary table of DEseq2 analysis, data transformed using relative log expression. [file Data_Sheet_1.PDF]

	log2FC	lfcSE	Pvalues	FDR
**Erysipelotrichaceae_unclassified**	**-2.6989**	**0.44432**	**1.25E-09**	**3.24E-08**
**Bacteria_unclassified**	**-4.7505**	**1.3915**	**0.000641**	**0.008328**
**Clostridiales_Incertae_Sedis_XIII_unclassified**	**-3.6813**	**1.164**	**0.001564**	**0.013555**
**Turicibacter**	**4.0964**	**1.4575**	**0.004945**	**0.025893**
**Terrisporobacter**	**4.2855**	**1.526**	**0.004979**	**0.025893**
**Acetatifactor**	**-1.8928**	**0.72441**	**0.008978**	**0.033075**
**Lactobacillus**	**-3.2267**	**1.2549**	**0.010132**	**0.033075**
**Clostridium_sensu_stricto**	**5.3484**	**2.0813**	**0.010177**	**0.033075**
Romboutsia	2.2948	1.1072	0.038198	0.11035
Oscillibacter	1.1454	0.59451	0.054025	0.13001
Pseudoflavonifractor	1.986	1.035	0.055003	0.13001
Clostridiales_unclassified	-2.0826	1.2505	0.095819	0.20761
Faecalibacterium	1.3574	0.92737	0.14327	0.28654
Lactococcus	0.90147	0.72305	0.21249	0.37071
Porphyromonadaceae_unclassified	0.37136	0.29876	0.21387	0.37071
Lachnospiraceae_unclassified	-0.16353	0.16983	0.3356	0.54534
Ruminococcaceae_unclassified	-0.15484	0.23234	0.50512	0.77254
Blautia	-0.52735	0.86998	0.54441	0.78637
Akkermansia	0.073205	0.20827	0.72522	0.92707
Asaccharobacter	-0.23341	0.70612	0.74098	0.92707
Clostridium_IV	0.32162	1.1639	0.7823	0.92707
Parasutterella	0.10211	0.37331	0.78444	0.92707
Clostridia_unclassified	-0.13915	1.0694	0.89648	0.97655
Clostridium_XlVb	-0.0723	0.9602	0.93998	0.97655
Clostridium_XVIII	-0.03133	0.70991	0.9648	0.97655
Bacteroides	-0.03562	1.2117	0.97655	0.97655
